# Stress Hyperglycemia in Critically Ill Patients: Insight Into Possible Molecular Pathways

**DOI:** 10.3389/fmed.2019.00054

**Published:** 2019-03-27

**Authors:** David Bar-Or, Leonard T. Rael, Robert M. Madayag, Kaysie L. Banton, Allen Tanner, David L. Acuna, Mark J. Lieser, Gary T. Marshall, Charles W. Mains, Edward Brody

**Affiliations:** ^1^Trauma Research Laboratory, Swedish Medical Center, Englewood, CO, United States; ^2^St. Anthony Hospital, Lakewood, CO, United States; ^3^Penrose Hospital, Colorado Springs, CO, United States; ^4^Wesley Medical Center, Wichita, KS, United States; ^5^Research Medical Center, Kansas City, MO, United States; ^6^Medical City Plano Hospital, Plano, TX, United States; ^7^SomaLogic Inc., Boulder, CO, United States

**Keywords:** sepsis, Warburg effect, hyperglycemia, glycolysis, oxidative phosphorylation, dipeptidyl peptidase IV

## Abstract

Severe sepsis, systemic inflammatory response syndrome (SIRS), and traumatic brain injury are frequently associated with hyperglycemia in non-diabetic patients. In patients suffering from any of these conditions, hyperglycemia at admission to an intensive care unit (ICU) is directly correlated with increased mortality or morbidity. Although there was initial enthusiasm for insulin treatment to blood glucose levels below 110 mg/dL in these patients, recent understanding suggests that the potential for hypoglycemic complications make this approach potentially dangerous. More moderate glucose control seems to be more beneficial than the aggressive glucose lowering initially suggested. An important publication has shown that hyperlactatemia accompanying hyperglycemia could be the real culprit in bad outcomes. This suggests that coupling moderate glucose lowering with therapeutic agents which might treat the underlying metabolic disturbances in these conditions may be a better strategy. The key metabolic disturbance in these three conditions seems to be persistent glycolysis as an energy source even in the presence of adequate tissue oxygenation (the Warburg Effect). We look at recent advances in understanding aerobic glycolysis and possibly the action of DPP4 on incretins resulting in insulin dysregulation and suggest key metabolic pathways involved in hyperglycemia regulation.

## Introduction

Severe sepsis, systemic inflammatory response syndrome (SIRS), and traumatic brain injury (TBI) are conditions associated with significant morbidity and mortality. Hyperglycemia is often a consequence of these three related conditions. Although the first steps in response to severe infections (sepsis), severe tissue damage (SIRS), and brain injury subsequent to trauma (TBI) vary, the later steps, which lead to morbidity, multi-organ failure, and death, seem to be very similar (1).

In the initial phases of these three conditions, there is a very strong inflammatory response, with high levels of IL-1β, TNFα, and IL-6, among other cytokines and chemokines, being secreted by M1-type macrophages and others. In this initial pro-inflammatory stage of these critical illnesses, very high levels of blood glucose (hyperglycemia) are often observed in these patients. Hyperglycemia, even in non-diabetic patients, is a hallmark of these conditions in their initial phase but is also a prognostic indicator, with a general correlation between glucose blood levels and the outcomes of morbidity and death (2, 3). Glycemic control in the critically ill also affects the immune system with general attenuation of immune function which might avoid unnecessary inflammation in TBI but could prove disastrous in sepsis (4). Here we discuss molecular mechanisms leading to hyperglycemia in critically ill patients.

## Glucose Metabolism

Although a variety of tissue-specific and inducible transporters of the GLUT family are known, glucose import into normal resting cells is mediated mainly by the GLUT-1 glucose transporter. GLUT-4, for example, is stored intracellularly but can be transported to the cell membrane for glucose transport in an insulin-dependent series of steps. In the pro-inflammatory phase of critical illness, metabolic stress leads to glycogen breakdown, catecholamine, and adrenocorticotrophic hormone synthesis, glucagon synthesis, and insulin resistance, all of which contribute to the hyperglycemia often seen in this phase of the three critical illnesses noted above (5–7).

Of particular importance is the role of catecholamine release in sepsis and SIRS. Catecholamines, once thought to be primarily released from neuroendocrine cells, are now known to be synthesized and released from macrophages and leukocytes (8). This involvement plays a major role in increasing glucose production during acute inflammatory disease (9). An open question for many years was whether the hyperglycemia seen in sepsis (and by inference in other acute inflammatory diseases) was primarily due to the increased glucose production seen in septic tissues or was primarily a result of poor glucose clearance in these tissues. Although there are some instances in which decreased glucose clearance contributes to hyperglycemia in septic patients with normal levels of lactate (10), the most meaningful study showed that in severe sepsis, hyperglycemia was primarily due to increased production of glucose (11).

There has been an impetus for using insulin to treat the hyperglycemia frequently seen in severely ill patients in emergency rooms and intensive care units (ICUs). This impetus stemmed primarily from a publication reporting that intensive insulin therapy, which maintained blood glucose levels at or below 110 mg/dL, reduced morbidity and mortality among critically ill patients in a surgical ICU (12). Consequently, insulin therapy for critically ill patients essentially became a standard of care, but not every ICU group found equally successful outcomes. The group that published the 2001 article also later reported on intensive insulin therapy on critically ill patients in a medical ICU, in which morbidity but not mortality was reduced by this treatment (13). In the multicenter VISEP clinical trial, intensive insulin therapy (insulin infusion started at >200 mg/dL [glucose] to maintain 80–110 mg/dL) was compared with conventional insulin therapy (insulin infusion started at >110 mg/dL [glucose] to maintain 180–200 mg/dL) in 537 patients with septic shock (14). The trial was halted early since the rate of severe hypoglycemia ([glucose] < 40 mg/dL) and serious adverse events was higher especially in the intensive therapy group. The debate surrounding this issue continued, although the intensity was reduced when a study by the NICE-SUGAR study investigators was published in 2009 (15). These authors found increased mortality among adults subjected to intensive glucose control in the ICU but also that increasing the target glucose level to 180 mg/dL resulted in lower mortality. A recent review separated trials on surgical and medical patients and recommended a glucose target using insulin therapy of between 140 and 180 mg/dL. This mitigated both the increased mortality and morbidity associated with very high glucose levels and the dangers associated with hypoglycemia occasionally seen with intensive insulin therapy (16).

It seems to us that the key to understanding the nature of this problem and suggesting new lines of reasoning for its resolution comes from an article published in 2012. In this article, Green et al. (17) showed that hyperglycemia in septic patients was associated with increased mortality risk only when the hyperglycemia was associated with simultaneous elevated concentrations of lactate in the blood. Glucose elevation did not pose an increased risk if lactate levels were not also elevated. In terms of causality, this places the problem squarely at the doorstep of lactate, which raises a whole new set of reflections on how one might mitigate this risk with therapies other than insulin. Lactate, of course, is the end product of glycolysis, and one of the hallmarks of sepsis, SIRS, and TBI is an increase in aerobic glycolysis (also known as the Warburg Effect) at the expense of the tricarboxylic acid cycle (also known as the Krebs cycle), which is normally responsible for much more efficient use of glucose in aerobic synthesis of ATP in mitochondria (18).

Of course, lactate production is somewhat organ specific and is usually associated with skeletal muscle oxygen deficit in normal physiology. Moreover, the liver can utilize blood lactate to resynthesize glucose (a series of reactions known as the Cori cycle) which can then be re-transported to muscle and other tissue. The Cori cycle, nonetheless, leads to a net expenditure of four molecules of ATP for every molecule of glucose converted to lactate and then re-synthesized, so that it cannot be used for a long period of time. Lactate production seems to be the most pronounced in those organs the most implicated in sepsis (19), which, again, argues against the muscle-liver axis playing a major role in net lactate production in sepsis and SIRS.

There is a large body of literature showing how increased lactate production can lead to decreased glucose utilization and, therefore, hyperglycemia. Hyperlactatemia in rats leads to decreased expression of the aforementioned GLUT-4 glucose transporter and decreased glucose uptake in muscle cells (20). Also in rats, lactate induces insulin resistance in skeletal muscle by inhibiting muscle glycolysis and impairing insulin signaling (21). These animal studies were followed by a number of studies in humans showing similar changes during critical illness (9, 11, 22).

## Metabolic Changes in Hyperglycemia

**Figure 1:**
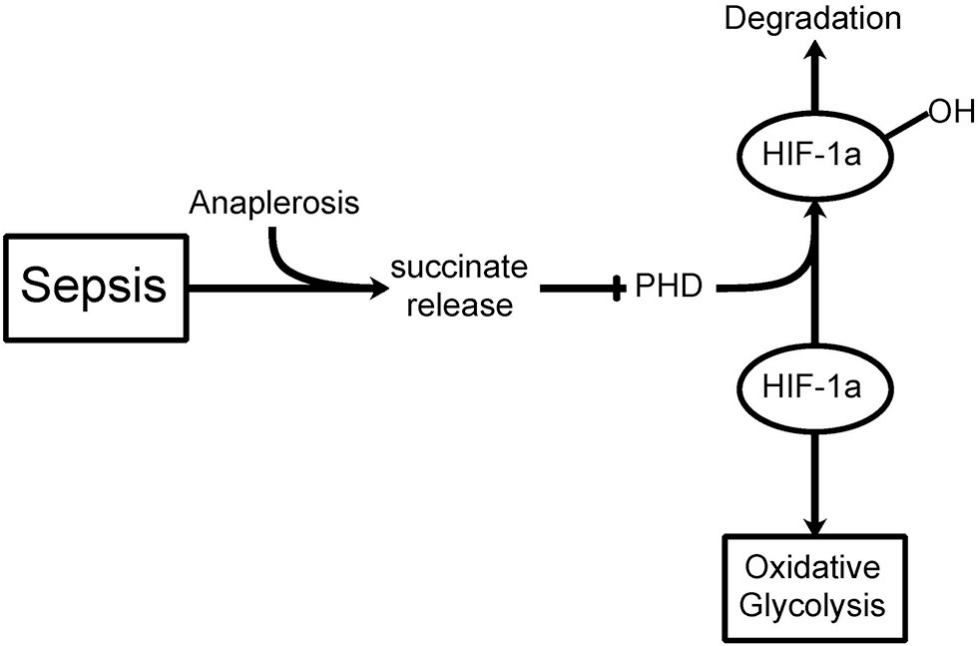
The Warburg Effect in sepsis. During septic shock, a build-up in mitochondrial succinate levels due to anaplerosis (glutamine transformation to α-ketoglutarate, a succinate precursor) is observed resulting in release into the cytoplasm. Prolyl hydroxylase (PHD) hydroxylates HIF-1α leading to its degradation. If PHD is inhibited by succinate release, then HIF-1α can inhibit the TCA cycle thereby favoring oxidative glycolysis (i.e., Warburg Effect) even when oxygen levels are sufficient.

If hyperglycemia is at least partially consequent to a shift in glucose metabolism and insulin resistance, then what does that imply for the treatment of hyperglycemia in critically ill patients? Although insulin treatment may constitute part of the therapy, is it not better to try insulin treatment in combination with new therapies that could possibly redirect the altered glucose metabolism in these cells? Recent advances in understanding the role of aerobic glycolysis in critically ill patients suggest novel mechanisms contributing to hyperglycemia and, as a result, novel approaches to therapy. One of the first important recent findings was the identification of increased succinate in macrophages stimulated by the pro-inflammatory compound lipopolysaccharide (LPS) (23). These authors conducted a thorough metabolomic study of this inflammatory pathway and confirmed the stimulation of glycolysis and inhibition of oxidative phosphorylation. Succinate was elevated some 30-fold during LPS stimulation. Succinate is transported from the mitochondria (where most of the excess is formed through anaplerosis via stimulation of glutamine transformation to α-ketoglutarate, the precursor to succinate in the TCA cycle) to the cytoplasm, where it inhibits prolyl hydroxylase, the enzyme that acts on HIF-1α and leads to its normal fast protein turnover (Figure 1). Inhibiting prolyl hydroxylase stabilizes HIF-1α and leads to aerobic glycolysis (the Warburg Effect) (24, 25).

Before we investigate more deeply into the hyperglycemia and hyperlactatemia observed in critically ill patients, we ask whether it is justifiable to treat hyperglycemia (and hyperlactatemia) as one entity in sepsis, SIRS, and TBI. The literature is somewhat confusing on this point. We have already mentioned that the original correlation of hyperglycemia and increased mortality came from patients treated in a surgical ICU and were often cardiac or thoracic surgical patients (12). When the same group reported on patients treated in a medical ICU, they found that intense insulin therapy to lower glucose levels decreased morbidity but not mortality (13). A meta-analysis of patients from five different ICUs (medical, cardiothoracic surgery, cardiac, surgical, and neurosurgical) in one large medical center showed differences in mortality when patients were very hyperglycemic among the five units. Laird and colleagues (26) found that early hyperglycemia ([glucose] >200 mg/dL) in trauma patients led to increased mortality rates independent of the type of injury sustained.

TBI patients also have worse outcomes, including increased mortality, when they are severely hyperglycemic ([glucose] >200 mg/dL) on admission to an ICU (27, 28). A more recent study not only confirmed this relationship but also related the prognostic capacity of initial glucose levels to the prognostic capacities of both the initial Glasgow Coma Scale (GCS) score and the Apache II score. Interestingly, initial mean glucose concentration was a better prognostic indicator for survival than either GCS or Apache II scores. Nonetheless, there are some unique properties of glucose metabolism in the brain. The glucose transporter GLUT-1 is the major transporter of glucose for non-neuronal cells (astrocytes, microglia, and oligodendroglia) and is also part of the endothelial blood-brain barrier. The GLUT-3 transporter seems to be the major source of glucose transport into brain neuronal cells. During glycolysis, accumulated lactate is shuttled between non-neuronal cells and neurons, using two different monocarboxylate transporters (MCTs) to effect this: MCT1 in endothelial and non-neuronal cells and MCT2 in neurons (29). Although the precise metabolism of lactate in the brain is still somewhat controversial (30), it is not important for further consideration here. The contribution of increased lactate to poor outcomes in TBI and the consideration of the biochemical targets that might overcome such outcomes seem to be no different than for sepsis and SIRS.

## The Warburg Effect in Critical Illness

We now refocus on the metabolic reprogramming that constitutes the Warburg Effect and the accumulation of succinate in critically ill patients (18). In macrophages and dendritic cells stimulated by LPS, a variety of metabolic changes occur, all of which lead to the inflammatory response. Inducible nitric oxide synthase (iNOS) increases the levels of nitric oxide (NO), which in turn nitrosylates iron-sulfur proteins in the mitochondrial electron transport chain, leading to inhibition of oxidative phosphorylation. LPS also leads to stimulation of the mTOR pathway, which augments synthesis of HIF-1α, stimulating glycolysis (as does the stabilization of HIF-1α via the inhibition of prolyl hydroxylase by succinate, as mentioned above). A large number of studies have also shown that one of three pyruvate kinases, PKM2, controls the level of oxidative phosphorylation in cells and that inhibiting PKM2 activity in macrophages reduces LPS-induced inflammation [reviewed in (31)]. A component of traditional Chinese medicine, shikonin, is an inhibitor of PKM2 and protects mice from LPS-induced shock and sepsis (32, 33).

Recent developments in understanding the Warburg Effect in critical illness suggest other, newer possibilities. A quantitative modeling of the glycolytic pathway using metabolomics and known kinetic parameters for the very well-studied enzymatic steps that control this pathway has been carried out (34). Variations in fructose 1,6-bisphosphate (FBP) led these authors to look for different rate-limiting steps as a function of FBP. They have deduced, and experimentally tested, the idea that during aerobic glycolysis the enzyme glyceraldehyde 3-phosphate dehydrogenase (GAPDH), which oxidizes glyceraldehyde 3-phosphate (GA3P) to yield NADH and 1,3-bisphospho-glycerate (1,3-BPG), becomes rate limiting for glycolysis (it is not rate limiting when FBP concentrations are low). Although the context for this work (and the work to be described below) was the Warburg Effect in cancer cells, all available evidence suggests that the aerobic glycolysis seen in sepsis should follow the same metabolic rules.

## Dipeptidyl Peptidase IV, Inflammation, and Hyperglycemia

Dipeptidyl peptidase IV (DPP4), or CD26, is a peptidase that preferentially cleaves Xaa-Pro and Xaa-Ala dipeptides from the N terminus of proteins (35). DPP4 activity has been reported on the cell surface of immune and endothelial cells (36), as well as in blood serum as a soluble form (37). The main function of DPP4 is thought to be the modification of biologically active peptides, cytokines, and other cell-surface proteins for the purpose of regulating the immune response and cell differentiation (35).

**Figure 2:**
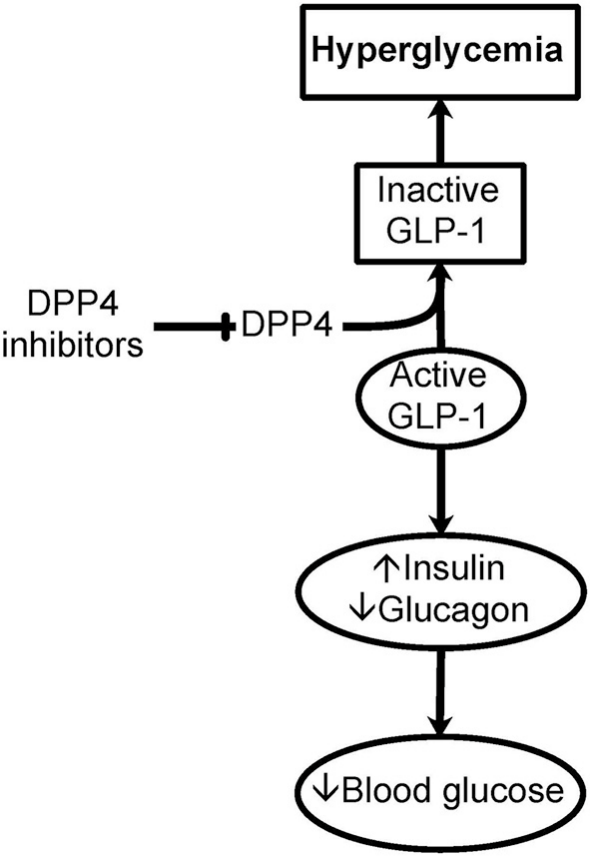
Importance of DPP4 in hyperglycemia. Dipeptidyl peptidase IV (DPP4), or CD26, is a cell surface and soluble peptidase that can cleave the first two N-terminal amino acids from specific proteins and peptides. DPP4 is up-regulated in immune and endothelial cells as a result of inflammation seen in critically ill patients. Of specific importance to hyperglycemia, DPP4 cleaves the N-terminus of glucagon-like peptide 1 (GLP-1). GLP-1 is important in the storage and regulation of blood glucose by promoting insulin and limiting glucagon release. This cleavage of GLP-1 causes the inactivation of GLP-1 contributing to hyperglycemia. Inhibitors of DPP4 could be important in glucose control in critically ill patients with hyperglycemia.

Of specific importance to the hyperglycemia encountered in critically ill patients is the action of DPP4 on incretins, specifically on glucagon-like peptide 1 (GLP-1) (38). Cleavage of His-Ala from the N-terminus of GLP-1 by DPP4 inactivates GLP-1 and its effect on insulin release stimulation and its inhibition of the release of glucagon, resulting in hyperglycemia (Figure 2). The same applies to inflammation.

The receptors for incretins are widely expressed in endothelial cells, vascular smooth muscle cells, monocytes, macrophages, and lymphocytes suggesting that GLP-1 could have direct effects on inflammation (38). Among the various effects of GLP-1, an important one is reduction of inflammation by reducing the levels of inflammatory mediators, decreased monocyte adhesion, reduction in the proliferation of macrophages and macrophage foam cell formation, and smooth muscle cell proliferation (38, 39).

In a large cohort of over 140,000 patients with type 2 diabetes, administration of DPP4 inhibitors is associated with an overall 75% increase in the risk of inflammatory bowel disease (40). However, in a separate study involving >100,000 patients with type 2 diabetes, combination therapy of DPP4 inhibitors with metformin resulted in a decreased risk of autoimmune diseases such as rheumatoid arthritis, lupus, psoriasis, multiple sclerosis, and inflammatory bowel disease (41).

DPP4 inhibitors that enable GLP-1 could have anti-inflammatory activities. To that effect, hyperglycemia is at least partially the result of increased DPP4 activity in these conditions.

## Conclusions

Stress hyperglycemia in critical illness may have some beneficial effects by supplying much-needed glucose to affected and hypo perfused tissue. However, this hyperglycemia has been associated with increased mortality and morbidity in surgical and medical patients. It appears that the combination of hyperglycemia and hyperlactatemia deserves specific attention in view of the clinical and molecular mechanisms described here.

## Author Contributions

DB-O and EB made contributions to conception and design, participated in drafting the article, and revised it critically for important intellectual content. LR, RM, KB, AT, DA, ML, GM, and CM participated in drafting the article and revised it critically for important intellectual content.

### Conflict of Interest Statement

EB is employed by SomaLogic, Inc. The remaining authors declare that the research was conducted in the absence of any commercial or financial relationships that could be construed as a potential conflict of interest.

## Abbreviations

SIRS: systemic inflammatory response syndrome
TBI: traumatic brain injury
GLUT: glucose transporter
ATP: adenosine triphosphate
LPS: lipopolysaccharide
TCA: tricarboxylic acid cycle
HIF-1α: hypoxia-induced transcription factor alpha
GCS: Glasgow Coma Scale
MCT: monocarboxylate transporter
iNOS: inducible nitric oxide synthase
NO: nitric oxide
mTOR: mammalian target of rapamycin
PKM2: pyruvate dehydrogenase kinase 2
FBP, fructose 1: 6-bisphosphate
GAPDH: glyceraldehyde 3-phosphate dehydrogenase
NADH: Nicotinamide adenine dinucleotide
DPP4: dipeptidyl peptidase IV
GLP-1: glucagon-like peptide 1.
